# Left-Sided Acute Appendicitis in a Patient With Situs Inversus Totalis

**DOI:** 10.7759/cureus.38105

**Published:** 2023-04-25

**Authors:** Hussain A Abdulla, Asma Alqaseer, Mohamed A Abushwemeh, Tareq Al Taei, Jassim Almehza

**Affiliations:** 1 Surgery, Salmaniya Medical Complex, Manama, BHR; 2 Radiology, Salmaniya Medical Complex, Manama, BHR

**Keywords:** acute abdomen, laparoscopic appendectomy, situs inversus totalis, left-sided acute appendicitis, acute appendicitis

## Abstract

Acute appendicitis is one of the most common reasons for presentation to the emergency department that requires an emergency appendectomy. Clinical presentation with abdominal pain in the left lower quadrant is very uncommon but can occur with a congenital left-sided appendix or right-sided long appendix. We report a rare case of a 65-year-old man with incidental finding of situs inversus totalis who presented with left lower quadrant abdominal pain. A CT scan of the abdomen confirmed the diagnosis of left-sided acute appendicitis, and the patient underwent laparoscopic appendectomy with an uneventful postoperative course.

## Introduction

Acute appendicitis is one of the most common causes of acute abdomen, accounting for approximately 6% of all emergency department visits [1]. Left-sided acute appendicitis is very uncommon and can happen in association with congenital abnormalities, such as situs inversus and midgut malrotation, or as an atypical presentation of the right-sided long appendix which projects into the left lower quadrant [2]. Situs inversus is a rare condition in which the orientation of asymmetric organs is a mirror image of normal anatomy. It may be partial, when only one of the abdominal or thoracic cavities is involved, or complete (situs inversus totalis), with transposition of both abdominal and thoracic organs. The diagnosis of left-sided acute appendicitis can be difficult and often delayed due to atypical clinical presentation [3]. We report an unusual case of situs inversus totalis with left-sided acute appendicitis managed by laparoscopic appendectomy.

## Case presentation

A 65-year-old Bahraini gentleman presented to our emergency department with a history of abdominal pain of one-day duration localized to the left iliac fossa. He also complained of anorexia, nausea, and vomiting. His medical history was significant for diabetes mellitus treated with oral hypoglycaemic agents. He was also on tamsulosin for benign prostatic hyperplasia. His surgical history was unremarkable. Physical examination revealed tenderness in the left lower quadrant associated with rebound tenderness.

**Figure 1:**
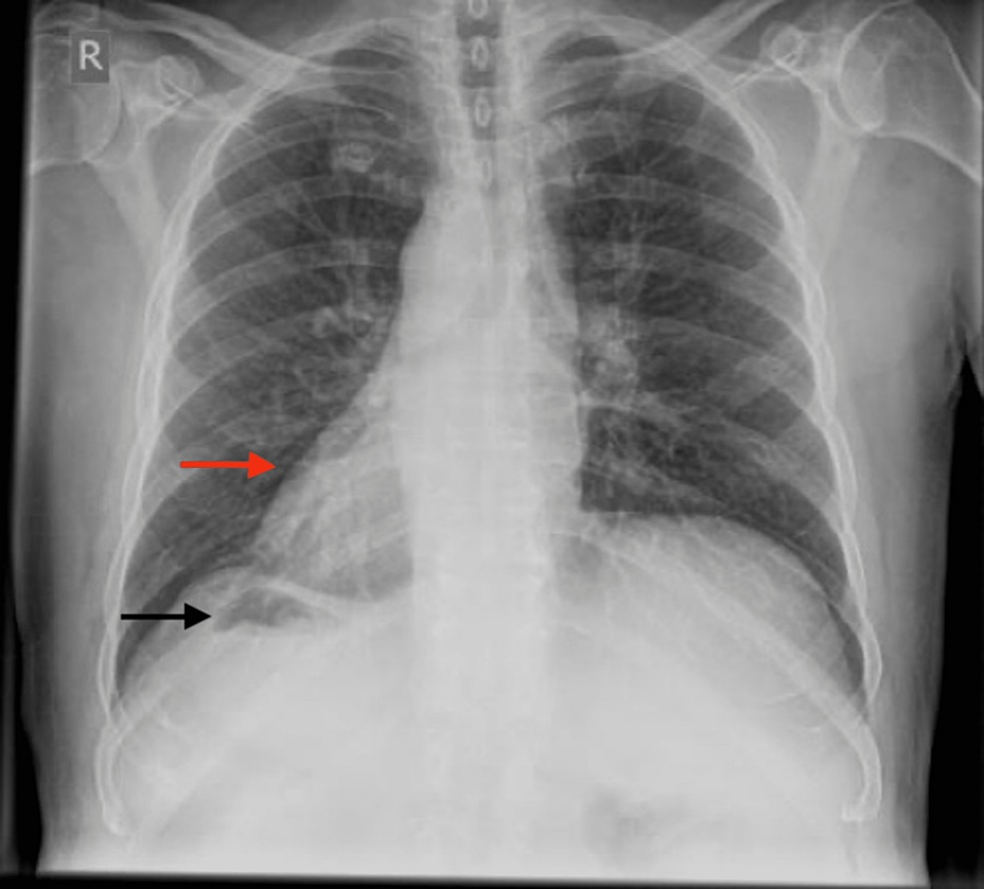
Chest X-ray shows dextrocardia (red arrow) and gastric bubble (black arrow) on the right side of the abdomen

Laboratory investigations showed leukocytosis (white blood cell count 10.4 x 109/L) with neutrophils accounting for 92% of the cell population. Other laboratory results, including biochemistry and urinalysis, were within normal limits. ECG showed a right axis deviation. A chest X-ray suggested the presence of dextrocardia, elevated left hemidiaphragm, and a right-sided gas shadow of the stomach fundus (Figure 1).

**Figure 2:**
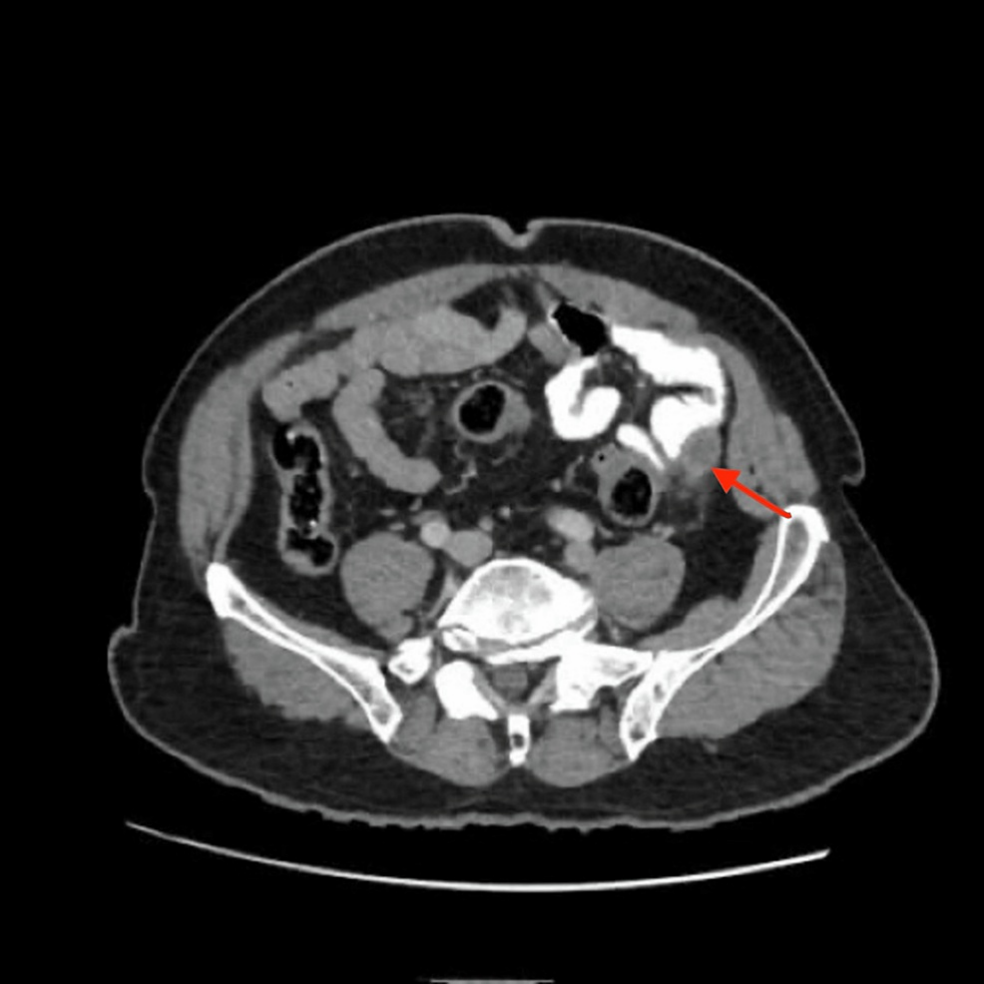
CT scan of the abdomen showing a swollen appendix in the left iliac fossa with adjacent fat stranding (red arrow)

**Figure 3:**
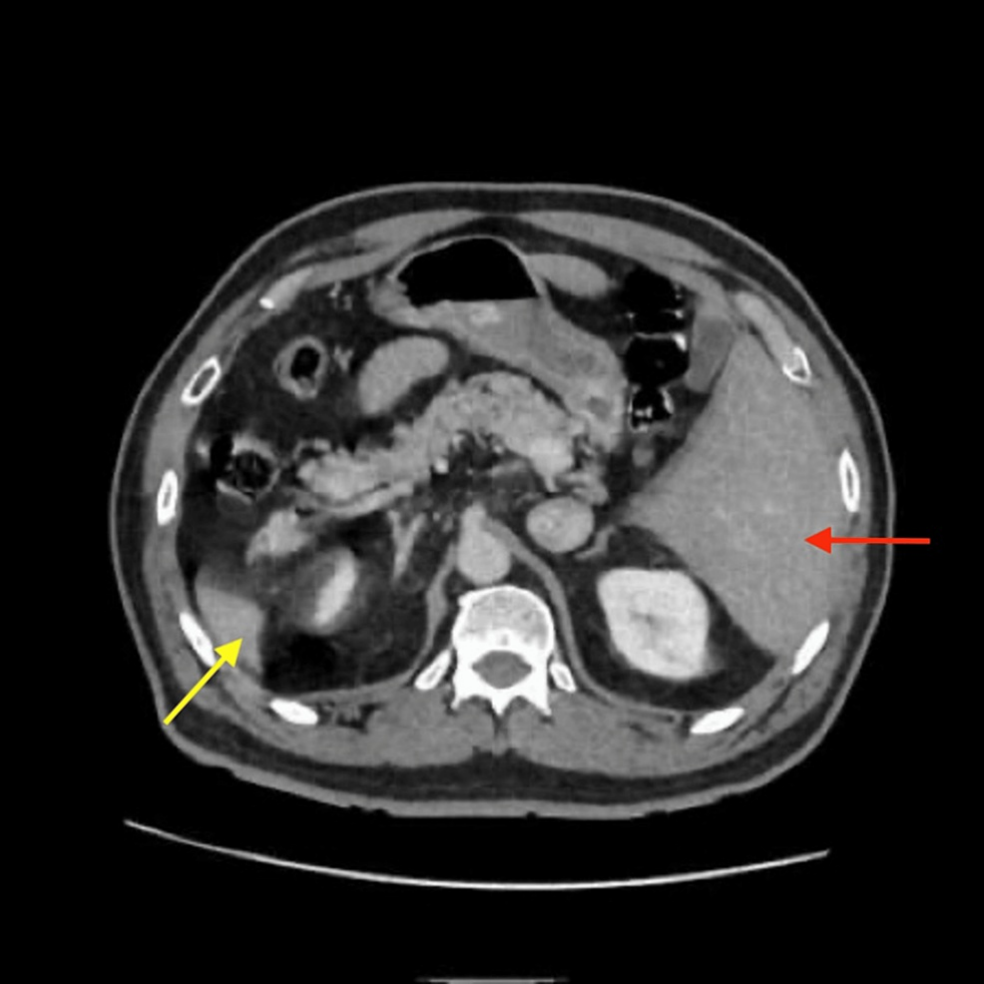
CT scan of the abdomen showing left-sided liver (red arrow) and right-sided spleen (yellow arrow)

A CT scan of the abdomen revealed a swollen left-sided appendix with thickening of the wall (maximal diameter 1.8 cm) and adjacent fat stranding (Figure 2) with transposed intra-abdominal organs (Figure 3), indicating situs inversus totalis with acute appendicitis.

**Figure 4:**
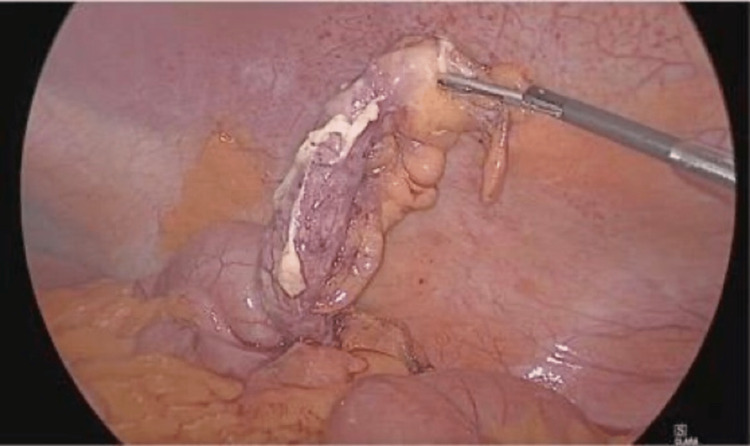
Laparoscopy showing left-sided acutely inflamed appendix

The patient underwent a laparoscopic appendectomy. We used the open Hasson technique to place the primary 11 mm trocar supraumbilically. Then, we placed the secondary 5 mm trocar at the level of the umbilicus on the left side of the abdomen. We introduced the last 5 mm trocar in the suprapubic region. Intraoperative findings were an inflamed, non-perforated, and non-gangrenous appendix with fluid collection in the left iliac fossa (Figure 4).

**Figure 5:**
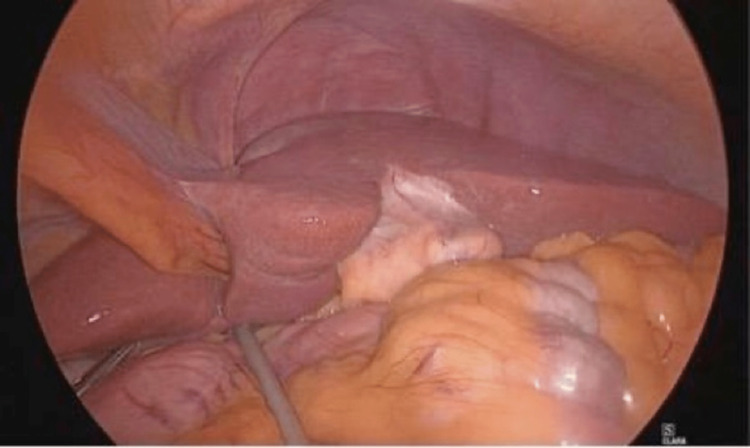
Laparoscopy showing evidence of the left-sided liver and gallbladder and right-sided stomach

Laparoscopic survey was performed, confirming situs inversus; the gallbladder and liver were left-sided, while the spleen and stomach were right-sided (Figure 5).

The estimated operative time was around 37 minutes. The patient was discharged on the second postoperative day without any complications. Pathology confirmed the presence of acute suppurative appendicitis.

## Discussion

Around a third of patients with acute appendicitis have abdominal pain in sites other than the right iliac fossa, due to significant variations in the location of the vermiform appendix [4]. However, acute appendicitis presenting with left lower quadrant abdominal pain is usually uncommon [2]. The differential diagnosis of left iliac fossa pain in adults is broad, including bowel obstruction, acute sigmoid diverticulitis, strangulated or incarcerated hernia, small bowel enteritis, Meckel's diverticulum, ruptured ovarian cyst or ectopic pregnancy, ureteric colic, acute epididymitis, psoas abscess, and acute appendicitis [1]. Left-sided appendicitis may be an unusual presenting feature of a long right-sided appendix extending into the left lower quadrant or of a mobile and redundant caecum [4,5]. It can also develop as a result of two types of congenital anomalies, which include midgut malrotation and situs inversus [3].

Malrotation is due to nonrotation or partial rotation of the intestines around the superior mesenteric artery, and the incidence varies from 0.03% to 0.5% in live births [1]. Situs inversus, a very uncommon entity, is a rare autosomal recessive congenital abnormality that occurs when there is a 270-degree rotation clockwise, resulting in complete transposition of all intra-abdominal organs [6]. The incidence of situs inversus totalis in the literature is 0.001-0.01% in the general population, and in patients with acute appendicitis, the incidence is 0.016-0.024% [2]. The diagnosis of left-sided acute appendicitis represents a clinical challenge and may be delayed due to its atypical clinical presentation. Although the abdominal viscera are displaced, the pain caused by left-sided acute appendicitis in the situs inversus may be felt in the right lower quadrant, which can cause misleading signs and symptoms [3].

Diagnosis of left-sided acute appendicitis in these patients can be made by means of physical examination, chest X-ray, ECG, USG, CT scan, and laparoscopy [4,6]. Physical examination may reveal right-sided heart sounds, left lower quadrant tenderness, palpable liver edge on the left side, and the right testicle lower than the left [2]. X-rays are not usually useful in diagnosing acute appendicitis, but the presence of dextrocardia and right-sided gastric bubble on chest X-ray, as in our case, can suggest situs inversus totalis. Our patient’s ECG showed right axis deviation. There has been increasing use of USG and CT scans for the diagnosis of acute appendicitis in the last few decades. USG is a widely used imaging modality to diagnose acute appendicitis, but it is limited in obese patients and those with overlying bowel gas, as well as being operator dependent [3]. On the other hand, a CT scan is extremely useful in the diagnosis of appendicitis in these patients because it outlines the abnormal location of the appendix, with a reported sensitivity of 90-98% [4]. In our case, we suspected left-sided acute appendicitis with situs inversus totalis based on the history, physical examination, and chest X-ray findings, and we conducted an abdominal CT scan to confirm the diagnosis.

After establishing the diagnosis of situs inversus totalis with left-sided acute appendicitis, the surgical treatment options are the same as those with an otherwise normally located appendix [3]. Laparoscopic appendectomy in situs inversus totalis was initially reported in 1998, but the technical aspects of the procedure were not described [7]. Our trocar sites were mirror image trocar positions of the routine laparoscopic appendectomy but with the right-handed surgeon using the left hand as the working hand. Therefore, the duration of the operation was 37 minutes, which is a little longer than a standard laparoscopic appendectomy. Handedness could influence the performance of operations in situs inversus totalis, and a left-handed operator could overcome this technical challenge more conveniently [8]. Nevertheless, laparoscopy is extremely helpful in the management of these cases, both by confirming the diagnosis and performing the surgery [2].

## Conclusions

Knowledge of differential diagnoses of left lower quadrant abdominal pain and having a high index of clinical suspicion is necessary to make the diagnosis of left-sided acute appendicitis. USG, CT scan, and diagnostic laparoscopy can play an important role in establishing an accurate and prompt diagnosis. Laparoscopic appendectomy in situs inversus totalis can be more difficult technically due to the mirror image anatomy of intra-abdominal contents and the use of the left hand as the dominant one in the case of a right-handed surgeon.
